# The Biological Impact of Some Phosphonic and Phosphinic Acid Derivatives on Human Osteosarcoma

**DOI:** 10.3390/cimb46050290

**Published:** 2024-05-15

**Authors:** Zakzak Khaled, Gheorghe Ilia, Claudia Watz, Ioana Macașoi, George Drăghici, Vasile Simulescu, Petru Eugen Merghes, Narcis Ion Varan, Cristina Adriana Dehelean, Lavinia Vlaia, Laurențiu Sima

**Affiliations:** 1 Pharmaceutical Technology, Faculty of Pharmacy, “Victor Babeş” University of Medicine and Pharmacy, 2nd Eftimie Murgu Square, 300041 Timisoara, Romania; khaled.zakzak@umft.ro (Z.K.); vlaia.lavinia@umft.ro (L.V.); 2 Formulation and Technology of Drugs Research Center, “Victor Babeş” University of Medicine and Pharmacy, 2nd Eftimie Murgu Square, 300041 Timisoara, Romania; 3 Department of Biology-Chemistry, Faculty of Chemistry, Biology, Geography, West University Timisoara, 16 Pestalozzi Street, 300115 Timisoara, Romania; gheorghe.ilia@e-uvt.ro (G.I.); vasile.simulescu@e-uvt.ro (V.S.); 4 Department of Pharmaceutical Physics, Faculty of Pharmacy, “Victor Babeş” University of Medicine and Pharmacy of Timisoara, 2nd Eftimie Murgu Square, 300041 Timisoara, Romania; 5 Research Center for Pharmaco-Toxicological Evaluation, “Victor Babeş” University of Medicine and Pharmacy Timisoara, 2nd Eftimie Murgu Square, 300041 Timisoara, Romania; macasoi.ioana@umft.ro (I.M.); draghici.george-andrei@umft.ro (G.D.); cadehelean@umft.ro (C.A.D.); 6 Department of Toxicology and Drug Industry, Faculty of Pharmacy, “Victor Babeş” University of Medicine and Pharmacy Timisoara, 2nd Eftimie Murgu Square, 300041 Timisoara, Romania; 7 Department of Physical Education and Sport, “King Mihai I” University of Life Sciences from Timisoara, Calea Aradului 119, 300645 Timisoara, Romania; petrumerghes@usvt.ro (P.E.M.); narcisvaran@usvt.ro (N.I.V.); 8 Department of Surgery I, “Victor Babeş” University of Medicine and Pharmacy, 300041 Timisoara, Romania; sima.laurentiu@umft.ro

**Keywords:** 2-carboxyethylphenylphosphinic acid, HaCaT and SAOS-2 cells, in vitro screening, anti-osteosarcoma

## Abstract

Osteosarcoma malignancy currently represents a major health problem; therefore, the need for new therapy approaches is of great interest. In this regard, the current study aims to evaluate the anti-neoplastic potential of a newly developed phosphinic acid derivative (2-carboxyethylphenylphosphinic acid) and, subsequently, to outline its pharmaco-toxicological profile by employing two different in vitro human cell cultures (keratinocytes—HaCaT—and osteosarcoma SAOS-2 cells), employing different techniques (MTT assay, cell morphology assessment, LDH assay, Hoechst staining and RT-PCR). Additionally, the results obtained are compared with three commercially available phosphorus-containing compounds (P1, P2, P3). The results recorded for the newly developed compound (P4) revealed good biocompatibility (cell viability of 77%) when concentrations up to 5 mM were used on HaCaT cells for 24 h. Also, the HaCaT cultures showed no significant morphological alterations or gene modulation, thus achieving a biosafety profile even superior to some of the commercial products tested herein. Moreover, in terms of anti-osteosarcoma activity, 2-carboxyethylphenylphosphinic acid expressed promising activity on SAOS-2 monolayers, the cells showing viability of only 55%, as well as apoptosis features and important gene expression modulation, especially *Bid* downregulation. Therefore, the newly developed compound should be considered a promising candidate for further in vitro and in vivo research related to osteosarcoma therapy.

## 1. Introduction

Osteosarcoma (OS) is a malignant pathology that affects the bone system and accounts for over 3 million cases worldwide each year. Over the past few decades, advances in science have enabled the survival rate of patients to increase significantly, reaching over 50%. Nevertheless, OS is a global health concern [1]. Until recently, the conventional treatment for OS involved surgical intervention and amputation, but the survival rate was very low. Since the discovery of chemotherapeutic drugs, amputations have been prevented and the prognosis of the disease has improved. In medical research, gene therapy is a hot topic that offers new perspectives on the treatment of OS [2]. However, organophosphorus compounds have been recently investigated for their anti-osteosarcoma activity showing good anti-proliferative results through inhibition of a specific protease (neutral endopeptidase) responsible for carcinogenesis [3].

Phosphonic acids, phosphic acids and their derivatives represent two classes of phosphorus derivatives [4]. The phosphonic acids and their derivatives, one of the important categories of organophosphorus compounds, are characterized by several specific aspects, such as (i) a phosphorus atom bonded to two hydroxy groups; (ii) one P=O double bond; and (iii) one carbon atom [5]. Their applications are well presented by Sevrain et al. [6]. The phosphonic acids are also present in [7]. Studies showed that up to a quarter of the phosphorus available in the ocean is related to the phosphorus species that contain one P–C bond, an aspect that highlights the significant impact of these compounds in natural biochemical processes [8].

Phosphinic acid compounds (phosphinates) are derivatives of hypophosphorous acid H2P(O)(OH), introduced in 1855 through the pioneering work undertaken by August Wilhelm Hofmann [9]. This class of organophosphorus derivatives found a lot of applications in biochemistry, biology and medicine [10,11], as building units in materials chemistry [12] and also as flame retardants [13].

**Figure 1:**
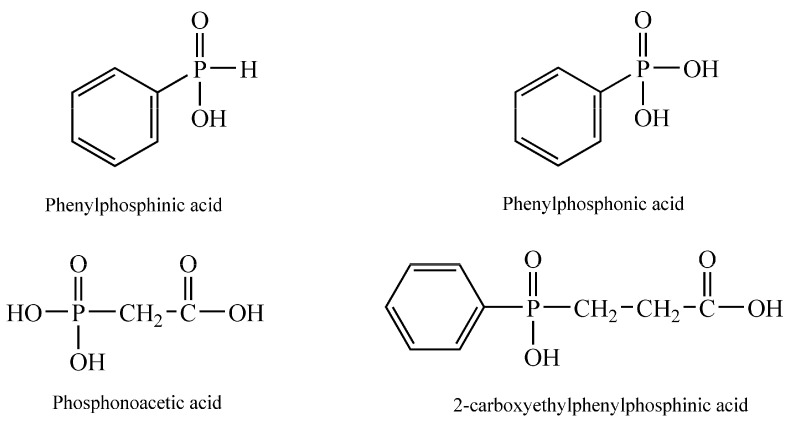
Chemical structures of phosphorus-containing test samples: phenylphosphinic acid, phenylphosphonic acid, phosphonoacetic acid and 2-carboxyethylphenylphosphinic acid.

Phenyphosphonic and phenylphosphinic acids and phosphonoacetic and 2-carboxyethylphenylphosphinic acids are part of two classes of phosphorus derivatives that are used as reagents or intermediates and have a lot of applications. The chemical structures of these compounds are presented in Figure 1.

Phenyphosphonic and phenylphosphinic acids were used for obtaining complexes [14], such as organic–inorganic hybrid materials obtained by the coordination of phosphonic (inic) ligands to metal ions, to obtain polymeric-related structures characterized by different dimensionalities [15] or as flame retardants [16,17]. Phosphonoacetic acid was used as an inhibitor of herpes simplex virus, [18], as a phosphorus source for microbial growth in a phosphate-independent manner [19] and also for obtaining heterobimetalic metal phosphonates [20]. 2-carboxyethylphenylphosphinic acid is one of the most promising reagents for obtaining metal–organic frameworks [21] or flame retardants for polymers [22].

Nevertheless, the implication of phosphorus-containing compounds in cytotoxic activity related to the anti-neoplastic potential of osteosarcoma was investigated a long time ago by Naviglio et al. who revealed that human osteosarcoma U2OS cell cultures manifested cell cycle inhibition with downregulation of cAMP and adenylate cyclase when exposed to inorganic phosphate [23]. Also, the same group investigated the role played by p53 in the mechanism of action developed by three different human osteosarcoma cell lines, as follows: (i) U2OS cells containing the wild-type p53; (ii) SAOS cells, a p53-deficient cell culture; and (iii) MG-63 cells expressing mutant p53 after exposure to inorganic phosphate. The results revealed that the human osteosarcoma apoptosis recorded was closely related to the p53-dependent pathway, as the cellular impairment induced by inorganic phosphate was only observed in U2OS cells (cells containing wild-type p53) and not in the other two cell lines expressing null p53 or mutant p53 [24]. Also, another study revealed a synergistic cytotoxic effect developed on U2OS cells when the cell cultures were exposed to both inorganic phosphate and different chemotherapeutics, such as doxorubicin and taxol [25], an aspect of great impact especially in the context in which the resistance to chemotherapy (doxorubicin and especially methotrexate-developed resistance) expressed by many osteosarcoma cell lines (HOS, MG-63, 143B, SAOS-2/±B) has been attested and correlated to drug transport impairment and specific mechanisms of action, such as RFC/SCL19A1 downregulation and DHFR overexpression, observed for methotrexate-resistant cell lines (HOS, MG-63, 143B, SAOS-2 and SAOS-2-B), while a multi-drug event endorsed by PgP upregulation was recorded for HOS-R/DOXO cells, a doxorubicin-resistant cell line [26].

As it is well known, organophosphorus derivatives have different toxicities, from low to high toxicity, such as chemical warfare agents (sarin, soman and so on) [27]; therefore, the current study aims to present the results related to the biocompatibility and anti-neoplastic activity of the previously mentioned compounds by using in vitro models, employing human keratinocytes (HaCaT cultures) and human osteosarcoma cells (SAOS-2).

## 2. Materials and Methods

### 2.1. Reagents

The following components were employed in the current study for culturing and analyzing cell lines: trypsin/EDTA solution, phosphate-buffered saline (PBS), dimethyl sulfoxide (DMSO), fetal calf serum (FCS), penicillin/streptomycin mixture of 100 U/mL penicillin and 100 μg/mL streptomycin and MTT reagent [3-(4,5-dimethylthiazol-2-yl)-2,5-diphenyltetrazolium bromide]; all of these reagents were purchased from Sigma Aldrich, Merck KgaA (Darmstadt, Germany). Phenylphosphinic acid (P1), phenylphosphonic acid (P2) and phosphonoacetic acid (P3) were also acquired from Sigma Aldrich. 2-carboxyethylphenylphosphinic acid (P4) was developed by our research group. Cell lines were cultured using Dulbecco’s Modified Eagle’s Medium—DMEM (P04-03550)—which was purchased from PAN Biotech GmbH (Aidenbach, Germany).

### 2.2. Preparation of 2-Carboxyethylphenylphosphinic Acid

The acid was prepared from dichloro(phenyl)phosphine and acrylic acid by a modified method than that mentioned in the literature [28]. To 7.16 g (0.040 moles) of dichloro(phenyl)phosphine, warmed to 80 °C, 6.75 g (0.094 moles) of acrylic acid was added drop-wise, simultaneous with the rise of temperature to 100 °C. The mixture was maintained for three hours at this temperature. Afterward, the mixture was added to 20 mL of water, stirred and cooled below 50 °C for a period of 3 h. The white slurry was cooled to 10 °C and filtered, and the solid was washed with water and dried, giving 7.5 g (86.0% yield) of 2-carboxyethyl(phenyl)phosphinic acid (I), a white solid; yield 80%; m.p. 157–159 °C; ^31^PNMR 36.9 ppm (literature 89.6% yield, white solid; m.p. 158–161 °C; ^31^PNMR-37.2 ppm). The melting point was determined using the capillary method. ^31^PNMR analysis was performed in DMSO-d6 at 25 °C on a Bruker DRX 400 MHz spectrometer (Billerica, MA, USA) (Figure S1).

### 2.3. Cell Culture

The cytotoxic potential of samples (P1, P2, P3 and P4) has been evaluated using the healthy human keratinocyte cell line HaCaT (catalog number: 300493) purchased from CLS Cell Lines Service GmbH as frozen vials and the osteosarcoma cell line SAOS-2 (catalog number: HTB-85^TM^), which was purchased from ATCC (American Type Cell Collection, Lomianki, Poland) as frozen vials. The cells were cultured in a specific culture medium (DMEM) supplemented with 10% FCS and 1% penicillin (100 U/mL)–streptomycin (100 µg/mL) mixture. During the experiment, the cells were maintained at standard temperature (37 °C) and 5% CO_2_.

### 2.4. Cellular Viability Assessment

To evaluate the impact of the analyzed compounds at the level of cellular metabolic activity by quantifying the mitochondrial reductase activity of the viable cells, the MTT method was applied. For this purpose, the cells were cultured in 96-well plates, in the number of 1 × 10^4^ cells/well. After reaching a suitable confluence, the cells were stimulated with P1, P2, P3 and P4 (1, 2.5 and 5 mM) for an interval of 24 h. After this time interval, the culture medium was renewed with 100 μL/well of fresh medium followed by the addition of MTT reagent (10 μL/well) for a period of 3 h. At the end of this time interval, 100 μL/well of solubilization solution was added for 30 min, and the microplates were maintained in a dark chamber at room temperature. To quantify the cell viability rate, absorbance was measured at a wavelength of 570 nm, using Cytation 5 (BioTek Instruments Inc., Winooski, VT, USA).

### 2.5. Cellular Morphology

The cell morphology of HaCaT and SAOS-2 cells was evaluated after 24 h of stimulation to assess the compounds’ cytotoxic potential. The impact on cellular morphology was evaluated by photographing the cells under bright field illumination using an Olympus IX73 inverted microscope (Olympus, Tokyo, Japan). Finally, the images were analyzed using cellSens Dimensions v.1.8 Software (Olympus, Tokyo, Japan).

### 2.6. Cytotoxicity Assay

To determine the necrotic-related events induced by test samples on both normal and tumorigenic cell lines—HaCaT and SAOS-2 cells, respectively—the lactate dehydrogenase (LDH) release method was employed. The principle of this method consists of quantifying the cytosolic enzyme LDH leaked into the extracellular medium, which can only be determined if the cellular wall is disrupted—indicative of necrosis [29].

The LDH test was performed by following the protocol provided by the manufacturer, as described in detail in a previously published article [30,31].

The absorbance was measured at 490 nm and 680 nm using a microplate reader—Cytation 5 (BioTek Instruments Inc., Winooski, VT, USA).

### 2.7. Nuclear Staining

To verify the effect induced by test samples (P1, P2, P3 and P4) at the nuclear level in HaCaT and SAOS-2 cells, Hoechst 33342 staining was employed by using a working solution of 1:2000 in PBS. The staining was realized 24 h post-exposure to test samples. To observe the nuclear changes that occurred after exposure to test samples, an Olympus IX73 inverted microscope (Olympus, Tokyo, Japan) was used, and pictures were taken under UV irradiation using the integrated DP74 digital camera. Further, the images obtained were analyzed using cellSens Dimensions v.1.8 Software (Olympus, Tokyo, Japan), and the apoptotic index was determined by quantifying the apoptotic cells relative to the total number of cells.

### 2.8. Real Time PCR Study

To investigate the impact of the four compounds (P1, P2, P3 and P4), the concentration of 5 mM was selected for applying the real-time reverse transcription–polymerase chain reaction (RT-PCR) method to the HaCaT and SAOS-2 cells to determine the impact of each compound on the expression levels of the genes *Bid*, *Bak*, *Bcl-xL*, *Bax*, *Bcl-2* and *Bad*.

#### 2.8.1. RNA Extraction and Quantification

For RNA extraction from the treated cells, the Quick-RNA Miniprep Kit (Zymo Research, Irvine, CA, USA) was utilized, following the manufacturer’s protocol. The concentration and purity of the extracted RNA were determined using a DS-11 spectrophotometer (DeNovix, Wilmington, DE, USA).

#### 2.8.2. cDNA Synthesis and RT-qPCR

The reverse transcription of extracted RNA to cDNA was carried out using the Maxima^®^ First Strand cDNA Synthesis Kit (Thermo Fisher Scientific, Inc., Waltham, MA, USA), adhering to the manufacturer’s guidelines. Quantitative real-time PCR (RT-qPCR) was subsequently performed using the Quant Studio 5 real-time PCR system (Thermo Fisher Scientific, Inc., Waltham, MA, USA), employing the Power SYBR-Green PCR Master Mix (Thermo Fisher Scientific, Inc., Waltham, MA, USA) for the amplification of specific gene sequences.

#### 2.8.3. Gene Targets and Analysis

Primer sequences specific to *Bid*, *Bak*, *Bcl-xL*, *Bax*, *Bcl-2* and *Bad* genes were used to ensure targeted amplification. The expression levels of these genes were normalized against a housekeeping gene (*GAPDH*). The oligonucleotides of the primers used in the present study were supplied by Thermo Fisher Scientific, Inc., Waltham, MA, USA, and their sequence is presented in detail in Table 1.

The analysis was conducted using the 2^−ΔΔCt^ method, which allowed for the determination of fold changes in gene expression relative to untreated control cells.

### 2.9. Statistical Analysis

The one-way ANOVA test followed by Tukey’s multiple comparisons post-test was employed to detect the statistical differences between multiple treatment groups, such as (i) the newly synthesized compound versus the commercially available compounds and (ii) human keratinocyte cell line HaCaT versus osteosarcoma cells SAOS-2. The data are expressed as mean ± standard deviation (SD) by using GraphPad Prism version 9.3.1 (GraphPad Software, San Diego, CA, USA, www.graphpad.com). The statistically significant differences between the analyzed data were marked with asterisks, as follows: * *p* < 0.05; ** *p* < 0.01; *** *p* < 0.001; **** *p* < 0.0001.

## 3. Results

### 3.1. Cellular Viability Assessment

In order to obtain an overview of the pharmaco-toxicological profile of the four samples, a healthy human keratinocyte cell line—HaCaT—and an osteosarcoma tumor cell line—SAOS-2—were used at the level of which the samples were tested for an interval of 24 h.

**Figure 2:**
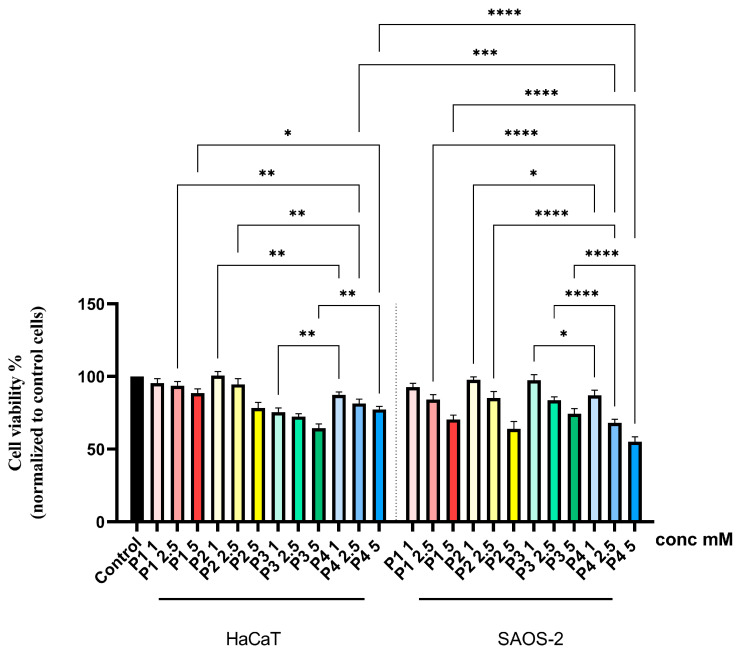
In vitro assessment of the cytotoxic effects of P1, P2, P3 and P4 (1, 2.5 and 5 mM) at the level of human keratinocytes HaCaT and human osteosarcoma cells SAOS-2 after 24 h of treatment. Results are presented as mean viability rate (%) of three independent experiments (*n* = 3) ± standard deviation (SD). Statistically significant differences between multiple treatment groups (different test samples and primary versus tumorigenic cells) were determined through one-way ANOVA and Tukey’s multiple comparisons post hoc test (* *p* < 0.05; ** *p* < 0.01; *** *p* < 0.001; **** *p* < 0.0001).

At the level of human keratinocytes, the four samples determined a concentration-dependent decrease in cell viability. Thus, in the case of all analyzed samples, the lowest viability was recorded at the highest concentration tested—5 mM. In the case of P1 and P2, the decrease in cell viability was not significant at the concentrations of 1 and 2.5 mM; instead, at the concentration of 5 mM, a value of approximately 88% and 78% was recorded, respectively. On the other hand, samples P3 and P4 determined a more significant decrease in cell viability, resulting in a minimum percentage of cell viability of approximately 64% for P3 and 77% for P4 (Figure 2). Regarding the effect of the compounds on the tumor cells, a similar trend was observed in the case of healthy cells of decreasing cell viability, but in this case, the cytotoxic effect was more pronounced. Thus, a significant decrease in the percentage of viable cells was observed, starting with the lowest tested concentration—1 mM—in the case of all compounds. However, the most intense cytotoxic effects were recorded at the concentration of 5 mM. Also, the most toxic compound, in this case, was P4, which caused a decrease in cell viability up to approximately 55% (Figure 2).

### 3.2. Cellular Morphology

For a more comprehensive evaluation of the cytotoxic profile, the impact on HaCaT and SAOS-2 cells’ morphology was examined.

**Figure 3:**
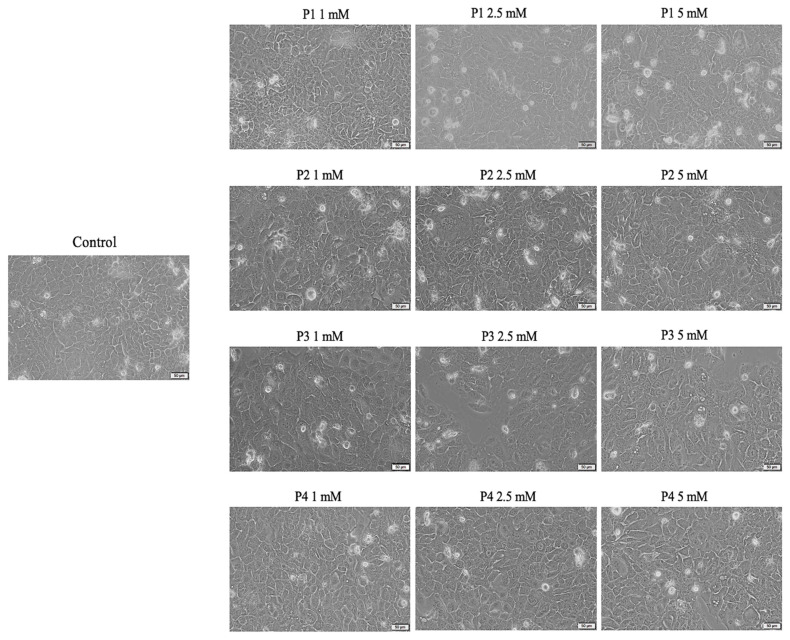
Morphological aspects of healthy human keratinocyte cell line HaCaT at 24 h post-exposure to P1, P2, P3 and P4 (1, 2.5 and 5 mM). Scale bars represent 50 µm.

As presented in Figure 3, the morphology of HaCaT cells did not present significant alterations, as compared to control cells, following treatment with P1, P2 and P4. However, the confluence of the HaCaT cell line decreased after exposure to concentrations of 2.5 and 5 mM of P3, yet the remaining cells developed a good interconnection pattern.

**Figure 4:**
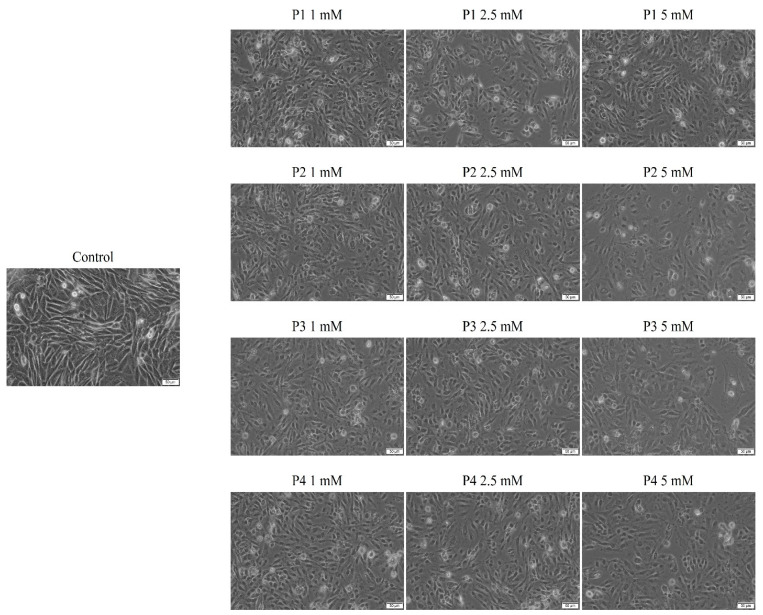
Morphological aspects of human osteosarcoma cells SAOS-2 after 24 h of treatment with P1, P2, P3 and P4 (1, 2.5 and 5 mM). Scale bars indicate 50 µm.

In the case of SAOS-2 cells, after a 24 h treatment, changes were observed regarding the shape of the cells, but also their confluence, in a dose-dependent manner. Thus, the most intense changes were recorded in the case of the concentration of 5 mM. Also, a difference was observed between the four samples in terms of the intensity of the morphological changes, the greatest impact being attributed to sample 4. In this case, changes in the cell shape were observed, the cells became round and detached from the plaque, they lost connections with neighboring cells, and there was a decrease in confluency, as well as a decrease in the number of cells attached to the plate. All these changes suggest a cytotoxic impact, the results being in accordance with those obtained after the cell viability test (Figure 4).

### 3.3. Cytotoxicity Assay

**Figure 5:**
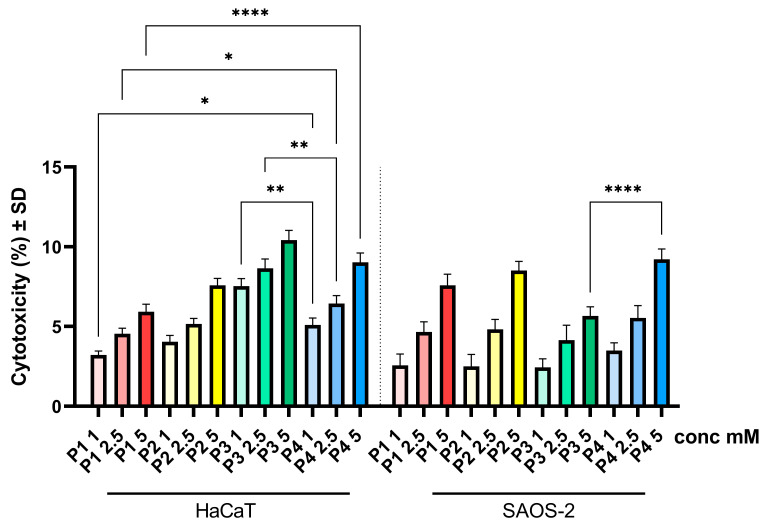
Cytotoxicity percentages of healthy human keratinocytes HaCaT and human osteosarcoma cells SAOS-2 after treatment with P1, P2, P3 and P4 (1, 2.5 and 5 mM). The LDH assessment was performed 24 h post-stimulation. Results are presented as mean cytotoxic rate of three independent experiments (*n* = 3) ± standard deviation (SD). Statistically significant differences between multiple treatment groups (different test samples and primary versus tumorigenic cells) were determined through one-way ANOVA and Tukey’s multiple comparisons post hoc test (* *p* < 0.05; ** *p* < 0.01; **** *p* < 0.0001).

Following the cell viability test, the LDH method was applied to determine cytotoxicity. Thus, in the case of both cell lines, HaCaT and SAOS-2 cells, no important cytotoxicity was recorded, as the cytotoxic percentage did not exceed 11%. However, an increase in the cytotoxic effect was observed in a dose-dependent manner. Thus, for HaCaT cells, the most pronounced cytotoxic effect was observed in the case of sample P3, followed by sample P4, with a percentage rate of 10.42 and 9.03%, respectively (Figure 5), whereas in the case of the osteosarcoma cell line—SAOS-2 cells—the highest percentage of cytotoxicity was observed for sample P4 (Figure 5).

### 3.4. Hoechst Staining

To analyze the impact of test samples (P1, P2, P3 and P4) at the nuclear level on HaCaT and SAOS-2 cell lines, Hoechst 33342 staining was performed.

**Figure 6:**
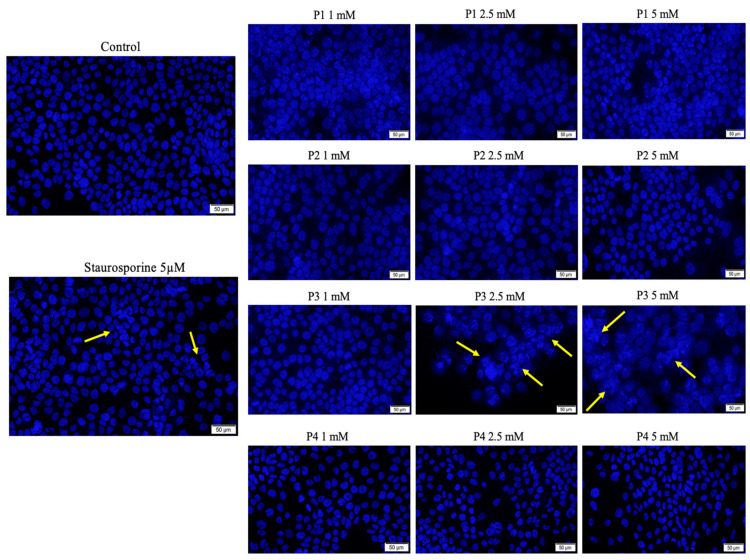
Cell nuclei staining of healthy human keratinocyte cell line HaCaT cells using Hoechst 33342 at 24 h post-exposure to P1, P2, P3 and P4 (1, 2.5 and 5 mM). Staurosporine (concentration of 5 μM) was used as positive control for inducing specific hallmarks of apoptosis. The apoptosis-related changes are marked by yellow arrows. Scale bars represent 50 µm. Cells were seeded at an initial density of 2 × 10^5^ cells/mL on a 38 mm^2^ well area. Data from one experiment representative of three are shown.

In the case of HaCaT cells, several apoptosis features were observed when the cells were treated with concentrations of 2.5 and 5 mM of P3, with the cells’ nuclei presenting specific signs of apoptosis-induced death, such as chromatin condensation and nuclear blebbing features, as indicated with yellow arrows in Figure 6. However, the cells treated with P1, P2 and P4 did not manifest important hallmarks of apoptosis, as depicted in the image collage (Figure 6). In this case, the apoptotic index (%) was below 30%, while P4 induced an apoptotic index above 60% when the highest concentration of 5 mM was employed (Figure S2).

**Figure 7:**
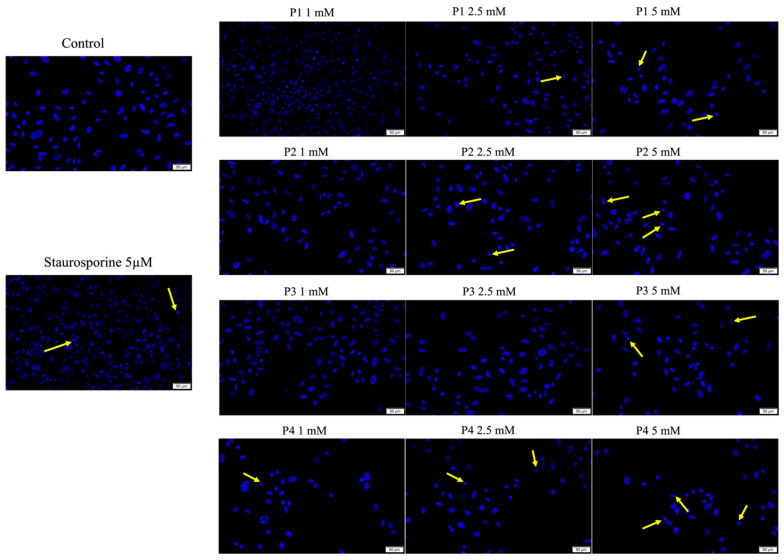
Cell nuclei staining of osteosarcoma cell line SAOS-2 cells using Hoechst 33342 at 24 h post-exposure to P1, P2, P3 and P4 (1, 2.5 and 5 mM). Staurosporine (concentration of 5 μM) was used as positive control for inducing specific hallmarks of apoptosis. The apoptosis-related changes are marked by yellow arrows. Scale bars represent 50 µm. Cells were seeded at an initial density of 2 × 10^5^ cells/mL on a 38 mm^2^ well area. Data from one experiment representative of three are shown.

The main changes observed in osteosarcoma cells were caused by concentrations of 2.5 and 5 mM in all tested samples. There were signs of chromatin condensation, the formation of apoptotic bodies, and a decrease in the number of nuclei in their cells, all of which are characteristics of apoptosis. The changes at the level of the nuclei were less pronounced in P3, with the cells exhibiting an apoptosis index of 29.1% for the concentration of 5 mM, while P4 caused the most significant changes, characterized by an apoptotic index of 52.6%, when the same concentration of 5 mM was used (Figure 7 and Figure S2).

### 3.5. Gene Expression

**Figure 8:**
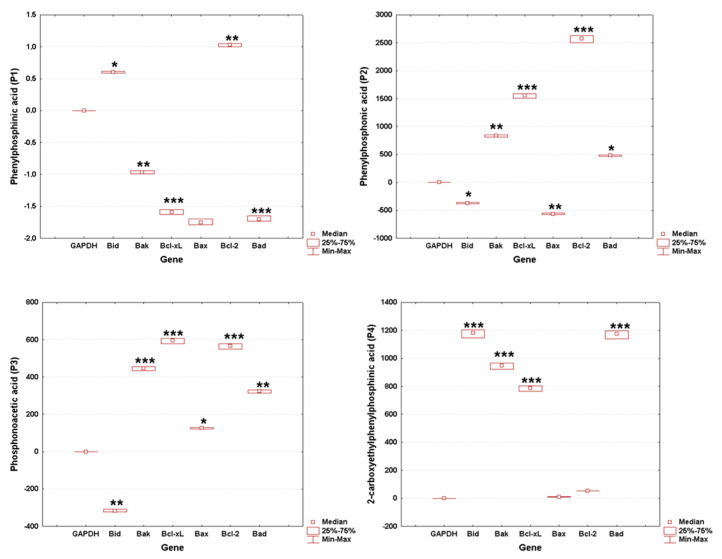
Effect of test sample (P1, P2, P3, P4) on apoptosis-related gene expression levels in HaCaT cells. Each treatment group was tested in triplicate across independent experiments to ensure data reliability and reproducibility. Gene expression was quantitatively analyzed and normalized to the GAPDH housekeeping gene. Statistical significance of changes in gene expression, when compared to the untreated control group, is highlighted with asterisks to denote levels of significance (* *p* < 0.05, ** *p* < 0.01, *** *p* < 0.001).

The results demonstrate differential modulation of the apoptotic pathway by each phosphorus-containing compound, reflecting their unique biochemical impacts on cellular mechanisms. P1 induced moderate gene downregulation that suggests a level of cellular impact but does not necessarily indicate severe toxicity, while P2 induced extreme variation in gene expression, raising concerns about potential toxicity, which could limit its biosafety profile (Figure 8).

**Figure 9:**
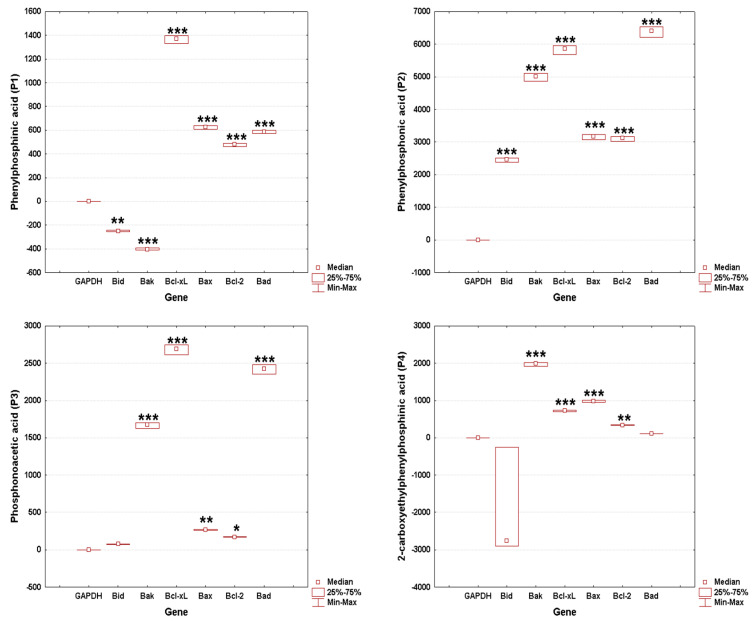
Effect of test sample (P1, P2, P3, P4) on apoptosis-related gene expression levels in SAOS-2 cells. Each treatment group was tested in triplicate across independent experiments to ensure data reliability and reproducibility. Gene expression was quantitatively analyzed and normalized to the GAPDH housekeeping gene. Statistical significance of changes in gene expression, when compared to the untreated control group, is highlighted with asterisks to denote levels of significance (* *p* < 0.05, ** *p* < 0.01, *** *p* < 0.001).

As shown in Figure 9, the significant downregulation in genes related to apoptosis and cell survival could be indicative of P1’s potential for anti-cancer activity, albeit not as pronounced as P4, in which case the extreme gene expression responses, particularly the downregulation of *Bid*, suggest a potent anti-cancer effect, aligning with the text’s observation of significant morphological changes and apoptosis induced by P4 in SAOS-2 cells. Also, P3 induced a substantial upregulation in apoptosis-related genes, which corroborates its effectiveness as an anti-cancer agent.

## 4. Discussion

As the biocompatibility and toxicological profile of a newly developed compound are considered among the most important aspects when referring to an upcoming prospective clinical use, the current study aims to provide a comprehensive biological evaluation of the recently synthesized product (P4) by discussing all the obtained results in comparison with three commercially available phosphorus derivatives (P1, P2 and P3) to deliver prospective data. To provide a complex biological profile, the present study is designed based on two different in vitro models, as follows: (i) a healthy cell line of human keratinocytes HaCaT to assess the biosafety level and (ii) a tumorigenic cell line consisting of human osteosarcoma cells SAOS-2 to evaluate the anti-neoplastic activity of the samples.

In vitro models were selected due to the fact that cell culture experiments are characterized by several important advantages when compared to in vivo models, such as performance enabled by cost-saving parameters and highly controllable conditions offering a good reproducibility feature [30,32].

The biological data obtained herein revealed several important aspects when the cell cultures (HaCaT and SAOS-2) were treated for 24 h with test samples, as follows: the cell viability assay showed that the healthy cell line of human keratinocytes (HaCaT) elicited the highest decrease rate when treated with P3, while the human osteosarcoma (SAOS-2) cell population was generally more affected compared to HaCaT cells, with the most important cell viability decrease recorded in the case of the P4 sample (Figure 2). Also, these data were in good agreement with those obtained for the cytotoxicity assessment via the LDH release method (Figure 5). In addition, the assessment of the cellular morphology showed that HaCaT cells treated with the P3 sample manifested the lowest cell confluence (Figure 3), while staining of cell nuclei also revealed apoptosis-related markers when HaCaT cells were exposed to the P3 sample for 24 h (Figure 6). When referring to the impact of the samples on the human osteosarcoma (SAOS-2) cell line, it was observed that sample P4 induced the most significant morphological alterations (Figure 4) and also high apoptosis features, such as chromatin condensation and apoptotic bodies (Figure 7). In addition, modulation of the apoptotic pathway by the P4 sample (Figure 9) corroborates the above-mentioned aspects by revealing important downregulation of *Bid*; this is indicative of the possible anti-neoplastic activity of this phosphorus-containing compound in SAOS-2 cells, as it disrupts the balance between pro-apoptotic and anti-apoptotic signals in SAOS-2 osteosarcoma cells, a phenomenon that is responsible for reducing cancer cell sensitivity to apoptotic signals, thus making the cells more resistant to treatment-induced cell death and promoting tumor survival.

These data revealed a good biosafety profile for the newly developed compound (P4 sample)—2-carboxyethylphenylphosphinic acid—that was even superior to the one from P3—phosphonoacetic acid—which is a compound already entered on the market and has long been widely used for anti-herpes simplex virus activity [33,34,35] and whose mechanism of action has recently been shown to be related to inhibitory activity at the level of DNA polymerases promoted by conjugation of the compound to the nucleobase [36].

Nevertheless, the data presented herein regarding the anti-osteosarcoma activity of the P4 sample (2-carboxyethylphenylphosphinic acid) are in good agreement with the data presented by Mizerska-Kowalska et al. [3], who investigated, for the first time, the anti-neoplastic potential of several organophosphorus compounds—derivatives of phosphonous acid–boranes—after obtaining favorable drug-likeness features for all the molecules investigated following the use of the SwissAdme tool. The prediction of physico-chemical properties and pharmacokinetic features of the test compounds was determined based on the Lipinski and Veber rules, as described in detail by Nicolov et al. [37]. However, Mizerska-Kowalska et al. [3] continued the study beyond the in silico evaluation by testing the organophosphorus compounds (derivatives of two genetically different osteosarcoma cell cultures: (i) HOS cells characterized by high tumorigenicity, invasion and migration activity and (ii) SAOS-2 cells that present medium tumorigenic potential, invasion and proliferation [38]), thus observing an anti-proliferative capacity, especially on HOS osteosarcoma cells, with the compounds acting as neutral endopeptidase inhibitor agents [3]. Moreover, several studies [23,24,25,39] have already endorsed the anti-osteosarcoma potential of high concentrations (ranging between 0.1 and 10 mM) of inorganic phosphate in human osteosarcoma cells. In addition to this, a study carried out by Pązik et al. [40] addressing the development of magnetite nanoparticles functionalized with different phosphonic moieties revealed that Na phosphonic salts, such as phenylphosphonate, 1,8-octanediphosphonate and ethylphosphonate, cannot induce oxidative stress in BJ cells; however, the compounds interfere with the metabolic activity of the cell monolayers, which was quantified through MTT assay. This type of cytotoxic effect through metabolic activity alteration was also observed in our study on SAOS-2 cells, as the viability rate of the cells pretreated with test samples was also quantified as a ratio of metabolic activity. The P4 sample induced significant metabolic activity arrest, as the cell viability rate of SAOS-2 cells was quantified at a percentage of approximately 55% when the highest test concentration (5 mM) was applied.

In addition, new research is employing a biocompatible and biodegradable phosphorus isomer—black phosphorus—which exhibits a unique molecular structure that further assures the optic and electronic characteristics to enable its biomedical applications for alternative therapies in osteosarcoma treatment, such as photothermal therapy, drug delivery and 3D printing [41,42,43], resorting to different approaches, such as the following: (i) the development of 3D-printed scaffolds reinforced with black phosphorus nanosheets for an exquisite treatment of osteosarcoma, providing the necessary tools for both photothermal ablation of osteosarcoma and bone regeneration under physiological conditions [44]; (ii) the use of black phosphorus quantum dots to obtain a drug delivery platform that could be further used for both chemotherapy and photothermal treatment of osteosarcoma [45] or development of an injectable chitosan-derived hydrogel containing black phosphorus nanosheets and doxorubicin for both chemo- and photothermal therapy [46]; and (iii) the design of black phosphorus nanosheet multifunctional composites to provide effective ablation of osteosarcoma by means of a photothermal approach [47].

Altogether, the data in the current study related to the cytotoxic effect obtained for P4 on human osteosarcoma SAOS-2 cells are promising for its subsequent use, due not only to its osteosarcoma anti-promoter potential but also due to its good biocompatibility features.

Nevertheless, the current study presents some limitations related to the human osteosarcoma cell line (SAOS-2) used herein, which is governed by medium aggressiveness in terms of tumorigenicity, such as medium invasion, colony-forming features, migration and proliferation activity [38], while other genotypically different cell lines exhibiting different tumorigenic characteristics (e.g., HOS cells that manifest high aggressiveness or U2OS cells that present low tumorigenicity but high migration and proliferation features) were not included in the study. Therefore, the anti-osteosarcoma potential of the newly synthesized compound (P4) can only be attributed to a moderately invasive pathology, characterized by the tumorigenic features of the SAOS-2 cell line.

## 5. Conclusions

The biological profile of our recently developed compound (P4) indicated a good biosafety profile when the HaCaT cells were exposed for 24 h at a maximum concentration of 5 mM. Also, the sample elicited promising anti-osteosarcoma activity under the same in vitro parameters (using the highest concentration of 5 mM for 24 h time exposure). In addition, the amalgamation of gene expression analyses with phenotypic assessments, encompassing cell viability, cytotoxicity, and morphological changes, provides a multifaceted evaluation of the biological impacts of the compounds P1, P2, P3 and P4. Notably, 2-carboxyethylphenylphosphinic acid (P4) demonstrates a compelling profile, characterized by pronounced anti-neoplastic properties in the SAOS-2 osteosarcoma cell line, coupled with a relatively benign impact on the HaCaT cell line. These findings, indicative of a favorable therapeutic index, position P4 as a candidate of significant interest for further investigation in the context of oncological therapeutics. The data underscore the necessity of employing a dual-pronged approach in preclinical evaluations, encompassing both healthy and diseased cell models, to holistically appraise the therapeutic viability of novel compounds. Future research endeavors should be directed towards elucidating the mechanistic underpinnings of P4’s cellular interactions, with an emphasis on long-term effects and potential clinical translatability.

## Figures and Tables

**Table 1:** The primer pairs employed in the current experiments.

Genes	Forward Sequence	Reverse Sequence
*GAPDH*	AAGGTGAAGGTCGGAGTCAAC	GGGGTCATTGATGGCAACAATA
*Bid*	CCTTGCTCCGTGATGTCTTTC	GTAGGTGCCTAGGTTCTGGT
*Bax*	GCCGGGTTGTCGCCCTTTT	CCGCTCCCGGAGGAAGTCCA
*Bcl-2*	CGGGAGATGTCGCCCCTGGT	GCATGCTGGGGCCGTACAGT
*Bak*	ATGGTCACCTTACCTCTGCAA	TCATAGCGTCGGTTGATGTCG
*Bcl-xL*	ATCCCCATGGCAGCAGTAAAGCAAG	CCCCATCCCGGAAGAGTTCATTCACT
*Bad*	CCCAGAGTTTGAGCCGAGTG	CCCATCCCTTCGTCCT

## Data Availability

The original contributions presented in the study are included in the article/Supplementary Materials, further inquiries can be directed to the corresponding author.

## Supplementary Materials

The following supporting information can be downloaded at: https://www.mdpi.com/article/10.3390/cimb46050290/s1, Figure S1. ^31^P NMR analysis by employing a Bruker DRX 400 MHz spectrometer. Figure S2. Apoptotic index (%) of immortalized human keratinocytes (HaCaT) and human osteosarcoma cells (SAOS-2) after treatment with test samples (P1, P2, P3, P4) at concentrations of 1, 2.5 and 5 mM for an interval of 24 h. One-way analysis of variance (ANOVA) was applied to determine the statistical differences followed by Tukey’s multiple comparisons test (* *p* < 0.05; ** *p* < 0.01; **** *p* < 0.0001).
