# Supplementary material for: The Biological Impact of Some Phosphonic and Phosphinic Acid Derivatives on Human Osteosarcoma
Source: Curr Issues Mol Biol. 2024 May 15;46(5):4815–31. doi: 10.3390/cimb46050290 (PMC11120618; doi:10.3390/cimb46050290)
Supplement: Supplementary file 1 [file cimb-46-00290-s001.zip › cimb-2968899-supplementary.pdf]

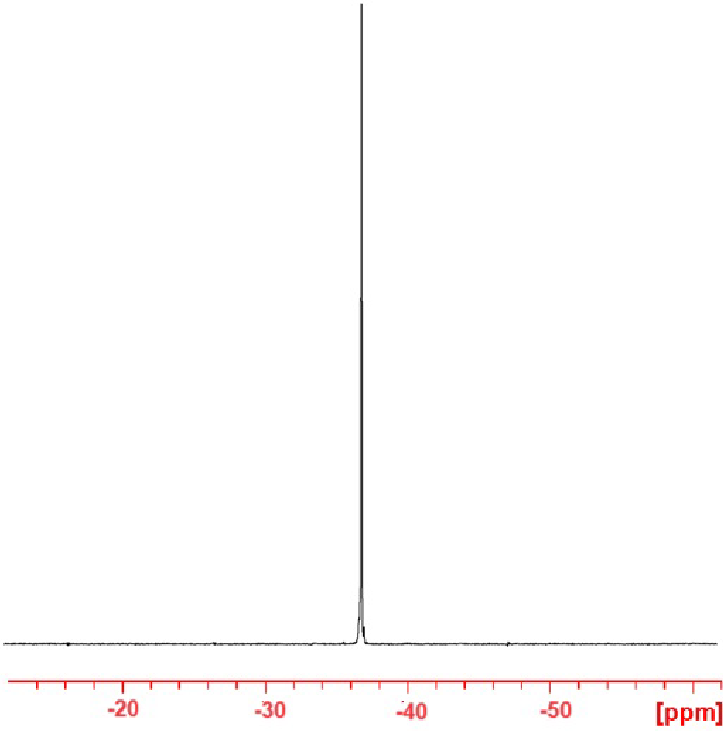

Figure S1. ^{31}P NMR analysis by employing a Bruker DRX 400 MHz spectrometer.

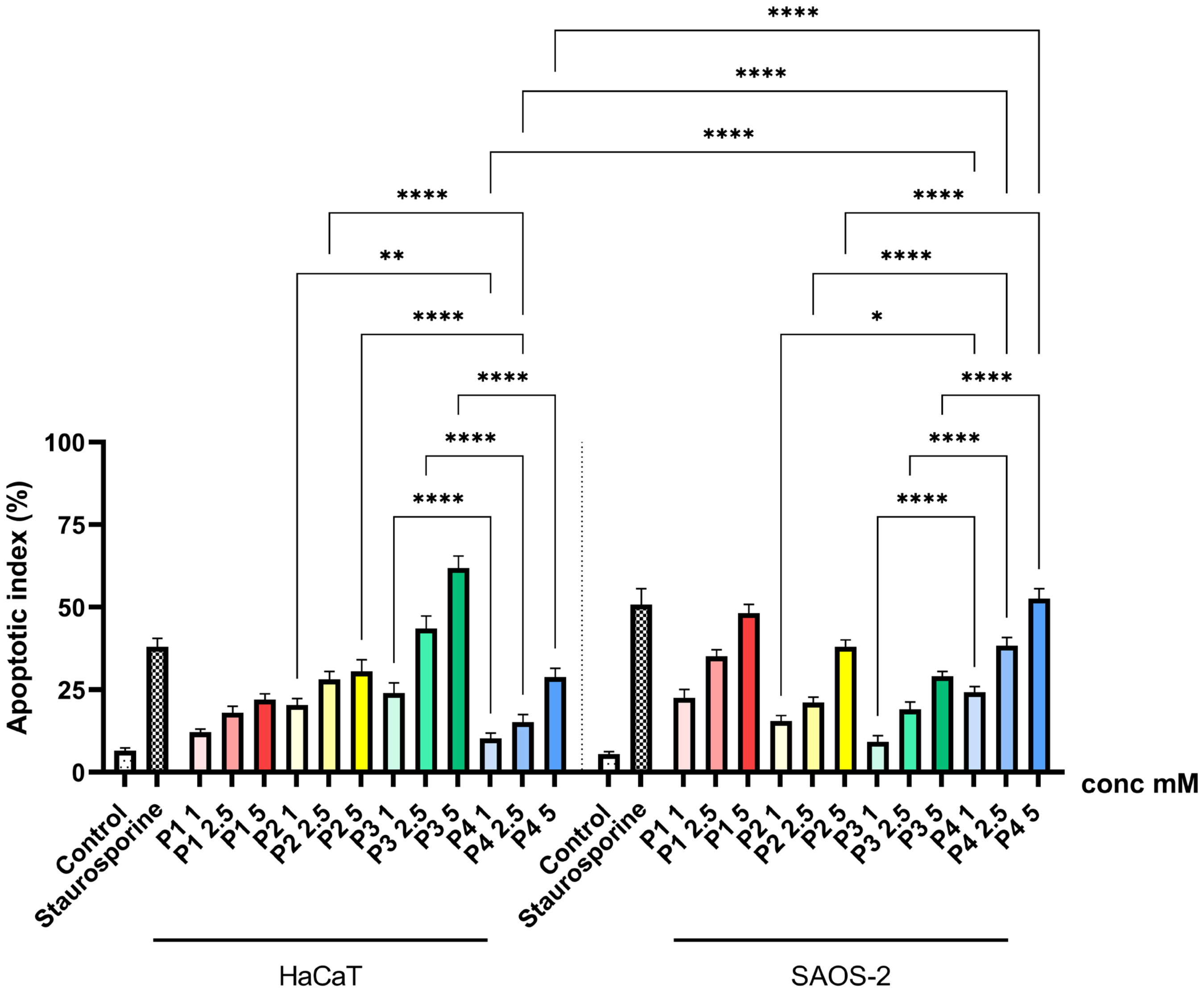

Figure S2. Apoptotic index (%) of immortalized human keratinocytes (HaCaT) and human osteosarcoma cells (SAOS-2) after treatment with test samples (P1, P2, P3, P4) at concentrations of 1, 2.5 and 5 mM for an interval of 24h. One-way analysis of variance (ANOVA) was applied to determine the statistical differences followed by Tukey's multiple comparisons test (* $p < 0.05$; ** $p < 0.01$; **** $p < 0.0001$).
